# Heterologous Immune Responses of Serum IgG and Secretory IgA Against the Spike Protein of Endemic Coronaviruses During Severe COVID-19

**DOI:** 10.3389/fimmu.2022.839367

**Published:** 2022-03-09

**Authors:** Wouter L. Smit, Sophie van Tol, Sanne van der Wal, Femke van Vulpen, Shannon la Grouw, Lenneke van Lelyveld, Gijs Limonard, Ailko Bossink, Gert-Jan Godeke, Sandhya Shrestha, Johan Reimerink, Dirk Eggink, Chantal Reusken, Michiel Heron, Steven Thijsen

**Affiliations:** ^1^Department of Medical Microbiology and Immunology, Diakonessenhuis Utrecht, Utrecht, Netherlands; ^2^Department of Medical Microbiology, University Medical Center Utrecht, Utrecht, Netherlands; ^3^Centre for Infectious Disease Control, National Institute for Public Health and the Environment (RIVM), Bilthoven, Netherlands; ^4^Department of Intensive Care, Diakonessenhuis Utrecht, Utrecht, Netherlands; ^5^Department of Pulmonary Diseases, Diakonessenhuis Utrecht, Utrecht, Netherlands

**Keywords:** SARS-CoV-2, COVID-19, spike protein, seasonal coronaviruses, immune imprinting

## Abstract

Defining immune correlates of disease severity is important to better understand the immunopathogenesis in COVID-19. Here we made use of a protein microarray platform to detect IgG- and IgA-reactive antibodies in sera and saliva respectively, and assess cross-reactivity between SARS-CoV-2 and endemic coronaviruses (eCoVs). IgG responses against the full protein of spike, but not the S1 subunit, were significantly higher in convalescent sera of patients with severe disease compared to mild disease and healthy controls. In addition, we detected reactivity of secretory IgA to eCoVs in saliva of patients with severe disease, not present in patients with moderate disease or seropositive healthy controls. These heterologous immune responses are in line with non-protective cross-reactivity, and support a potential role for immune imprinting in the pathogenesis of severe COVID-19.

## Introduction

Severe acute respiratory syndrome coronavirus 2 (SARS-CoV-2) causes a wide spectrum of symptoms known as COVID-19. Although a large majority of infections follows a mild or asymptomatic disease course, a proportion of individuals develop severe disease for which hospitalization and invasive ventilation is necessary. In risk groups of elderly or patients with pre-existing comorbidities, clinical presentation is complicated by respiratory failure, shock, or multiorgan failure, which is often fatal (1). Despite tremendous research efforts, immunopathogenic mechanisms or correlates of protection underpinning heterogeneity in host responses remain to be clarified.

Heterologous humoral and cellular immune responses induced by different related viruses lead to extremely complex host-pathogen interactions of which the outcome is unpredictable (2). Depending on the virus-host context, heterologous immunity may either be protective, ineffective or even counterproductive (3). For instance, during sequential influenza infections or after vaccination, cross-protective immune responses have clearly been observed (4). Yet, it is thought that antigenic drift over time could also elicited a non-productive memory recall response if the cross-reactive epitope is a non-dominant epitope (5).

Since the emergence of SARS-CoV-2, several studies have focused on the role of cross-reactivity and signs of cross-protectivity that may result from previous encounter with endemic human coronaviruses (eCoVs). Multiple studies have demonstrated evidence of memory T-cell induced cross-reactivity to SARS-CoV-2 in up to 50% of individuals with prior eCoV infections, predominantly directed at epitopes on the spike antigen (6–12). Similarly, sero-survey studies suggest presence of substantial humoral cross-reactivity between endemic eCoVs and SARS-CoV-2, explained by extensive sequence homology in the S2-subunit of spike protein (13, 14). Whether exposure to endemic eCoVs is associated with disease severity in COVID-19 or the clinical outcome, remains to be fully understood, although present data suggest that ﻿protective cross-neutralization might be unlikely (15, 16).

In this study, we assessed serological evidence of prior exposure to eCoV infections (HCoV-OC43, HCoV-HKU1, HCoV-229E and HCoV-NL63) and SARS-CoV-2 in a Dutch cohort of hospitalized patients. Anti-spike titers were correlated to COVID-19 severity. We also measured secretory IgA reactivity to SARS-CoV-2 and eCoVs in saliva to identify potential correlates of mucosal protection.

## Results

### Protein Microarray Analysis of IgG Responses Against Structural Viral Epitopes in Convalescent Sera Distinguishes Hospitalized Patients With Severe Disease From Those with Mild/Asymptomatic Disease

Patients admitted to the department of pulmonology or the intensive care unit (ICU) were enrolled as part of the SARS-CoV-2 immune response (SIR) study, upon confirmation of SARS-CoV-2 infection with a validated in-house PCR test. Enrolled patients were followed up over time. Sera and, if possible, saliva samples were collected at the day of hospital admission (acute phase), as well as 30 days and 3 months post-discharge (seroconvalescent phase). Concurrently, we included a group of healthy volunteers amongst hospital personnel, with or without a history of asymptomatic or mild COVID-19. Mild symptoms were recorded retrospectively using questionnaires. Proven history of exposure to SARS-CoV-2 infection was based on serostatus as measured by the Wantai Ab ELISA, a high-validity assay for detection of SARS-CoV-2 seroconversion in mild and severe disease through detection of total immunoglobulin antibodies binding the SARS-CoV-2 receptor binding domain (17).

The following groups were defined (**Supplementary Figure 1**): 1a) seronegative healthy controls, 2a) seropositive disease controls with a history of mild/asymptomatic SARS-CoV-2 infection, 2b) seropositive disease controls with a history of severe COVID-19 disease that required hospitalization and 3) patients during the acute phase of PCR-confirmed COVID-19 that were admitted to the pulmonary ward or ICU. All hospitalized patients contracted severe pneumonia which required non-invasive or invasive respiratory support.

We also included a small subset of seronegative healthy individuals of which we collected sera before and after immunization with the Pfizer/BioNTech vaccine (**Supplementary Figure 1**).

Amongst seropositive individuals with a history of mild/asymptomatic SARS-CoV-2 infection, 85% experienced flu-like symptoms, between 5-6 months prior to serum sampling, coinciding with the first wave of infections between March and May of 2020 in the Netherlands. 33% reported loss of smell or taste. 77% had a PCR-confirmed SARS-CoV-2 infection at the time, the remaining 23% was asymptomatic or had symptoms that did not meet the criteria for obligated PCR testing.

Amongst hospitalized patients, additional distinctions were made based on need for critical care (ICU) and outcome (**Supplementary Figure 1**). Patients at the pulmonary ward received a form of non-invasive respiratory support, which we referred to as moderate disease, whilst patients admitted to the ICU in need of invasive mechanical ventilation or other forms of critical care for complicated COVID-19 were referred to as severe disease. Secondly, we divided all hospitalized patients into those who survived and were discharged (termed non-fatal disease), and those who deceased during their stay in the hospital or up to one week after discharge (termed fatal disease). The main characteristics of each of these groups are presented in **Table 1**. As was previously reported, patients with fatal disease were significantly older and presented with a higher rates of comorbidity including diabetes, cancer, vascular or pulmonary disease (18). Patients with COVID-19 at the ICU had significantly higher C-reactive protein (CRP) levels, an acute phase reactant and a sensitive marker of an increased inflammatory state.

**Table 1:** Clinical characteristics of patients with COVID-19 disease.

	Healthy controls		Patients (per departement)		Patients (per outcome)		Pairwise comparisons		
	Seronegative	Seropositive	Moderate COVID-19	Severe COVID-19	Non-fatal COVID-19	Fatal COVID-19	p-value (seroneg VS seropos)	p-value (moderate VS severe)	p-value (non-fatal VS fatal)
Individuals (n)	34	26	34	25	40	19			
Male I female (n)	N/A	N/A	22 | 12	18 1 7	27 | 13	13 1 6		0.58	1
Age (years)	45± 14	37 ± 13	63 ± 16	66±10	58±12	77±8	0.06	0.82	<0.0001
BMI (Kg/m2)			28±7	31±6	30±7	27±7		0.19	0.08
Mean hospital stay (days)			10±16	20±6	13±11	22 ± 16		0.08	0.31
**Medical history**									
Diabetes mellitus			7 (21%)	9 (36%)	7 (18%)	9 (47%)		0.15	0.03
Hypertension			13 (38%)	10 (40)	12 (30%)	12 (63%)		1	0.023
Coronary disease			7 (21%)	8 (32%)	5 (13%)	10 (53%)		0.37	0.003
Pulmonary disease			14 (41%)	11 (44%)	12 (30%)	13 (68%)		1	0.01
Cancer			8 (24%)		4 (10%)	6 (32%)		0.17	0.02
**Medication**									
pre-existent immunomodulating drugs			6 (18%)	3 (12%)	6 (15%)	3 (16%)		0.72	0.45
**Inflammatory markers**									
CRP (mg/L)			76 ± 62	119 ±74	93 ± 70	93±72		0.04	1
leukocyte count (109/L)			7.1±4	8.3±4	7.8±4	7.2±3		0.44	0.81

Data are presented as mean with standard deviation or (%). P-values were calculated using one-way ANOVA with Šidák post-hoc correction for multiple pairwise comparisons or the Fisher’s exact test in case of categorical variables. N/A, not available; CRP, C-reactive protein.

Next, we measured IgG responses to various coronavirus antigens using a protein microarray as a multiplex serological assay (19). Our antigen panel consisted of spike protein from three SARS-CoV-2 variants of concern; the alpha (Pango lineage B.1.1.7) variant, beta variant (Pango lineage B.1.351) and the gamma variant (Pango lineage P.1). In parallel, we measured titers of antibodies to circulating *Alphacoronaviruses* (HCoV-229E and HCoV-NL63) and *Betacoronavirus* (HCoV-HKU1, HCoV-OC43, and the two other emerging human coronaviruses SARS-CoV-1 and MERS-CoV). Spike S1 subunit (denoted as spike S1) and the full-length trimerized spike protein (denoted as spike trimer or S-T) were analyzed separately in our assay.

**Figure 1:**
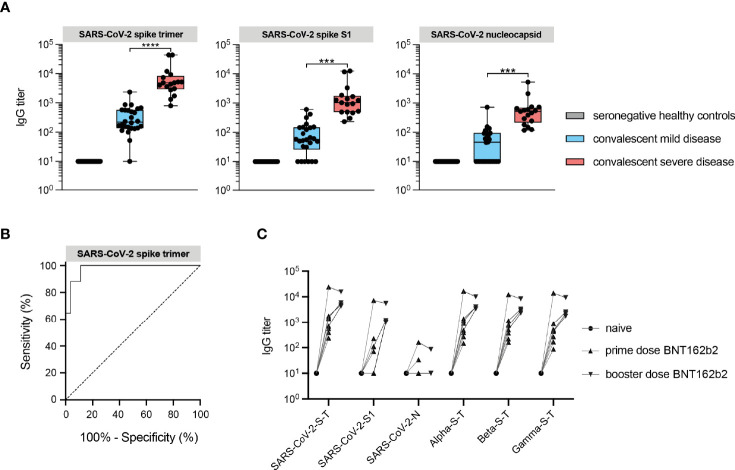
IgG responses against structural viral epitopes in convalescent sera distinguishes hospitalized patients with severe disease from mild/asymptomatic disease. **(A)** IgG antibody responses against SARS-CoV-2 recombinant spike and nucleocapsid structural proteins measured by protein microarray in convalescent sera from COVID-19 patients (red box), mild/asymptomatic disease controls (blue box) and unexposed healthy controls (gray box) Data are represented as the median titers (min and max value). **(B)** Receiver operating characteristic (ROC) curve analysis of protein microarray for discrimination between convalescent patients with severe COVID-19 requiring ICU care and those with mild/asymptomatic disease. **(C)** Vaccine-induced IgG antibody responses in seronegative healthy individuals vaccinated with spike mRNA vaccine (BNT162b2). SARS-CoV-2 refers to the original Wuhan-1 strain. ***P ≤ 0.001, ****P ≤ 0.0001.

Consistent with previously reports, IgG titers against spike and nucleocapsid protein (N) of SARS-CoV-2 were markedly elevated in convalescent sera of severely diseased patients compared to mild/asymptomatic disease controls (**Figure 1A**). High anti-spike responses against SARS-CoV-2 distinguished between a prior mild or severe disease course, with a sensitivity and a specificity of 94% and 96% respectively when a threshold of 1093 AU/ml was used (**Figure 1B**). The time period between symptom onset and serum sampling at convalescence was 4.5 months for severe patients and 5.5 months for mild disease controls on average (**Supplementary Figure 1**). This did not explain the observed difference in IgG titers, since this difference was still present in the subset of individuals of mild and severe disease in which these periods overlapped. In addition to assessment of natural immunity, we validated our assay by using sera to detect vaccine-induced responses in an additional small set of seronegative healthy individuals (also hospital employees) who were vaccinated with BioNTech/Pfizer (BNT162b2). As expected, robust IgG responses to SARS-CoV-2 spike protein were detected after the first dose. This effect was augmented after the booster and in response to all variants of concern (**Figure 1C**). For reasons not fully understood, two individuals showed a mild increase in antibodies that reacted with the nucleocapsid protein, a finding that has also been reported in another study of seronegative individuals receiving BNT162b2 (20). However, the observed responses to spike proteins demonstrate the utility of the microarray platform for parallel detection of multiple protein-specific immune responses.

### IgG Responses Against Spike Protein of eCoVs Are Induced in Convalescent Sera of Severe COVID-19 Patients

**Figure 2:**
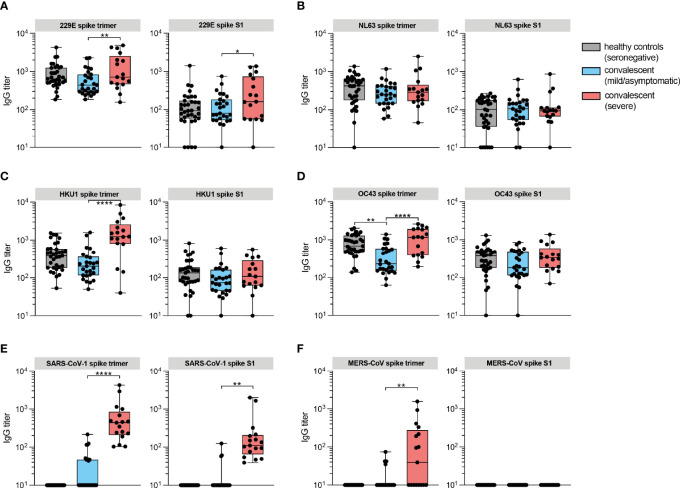
IgG responses against spike protein of eCoVs are induced in convalesced patients with severe COVID-19. **(A)** IgG antibody titers against recombinant trimeric spike proteins and monomeric S1 subunits of eCoV 229E measured by protein microarray in convalescent sera from COVID-19 patients (red box), mild/asymptomatic disease controls (blue box) and unexposed healthy controls (gray box). **(B)** Same as in A for eCoV NL63. **(C)** Same as in A for eCoV HKU1. **(D)** Same as in A for eCoV OC43. **(E)** Same as in A for SARS-CoV-1. **(F)** Same as in A for MERS-CoV. Data are represented as the median titers (min and max value). Significance (one-way ANOVA) *P < 0.05, **P < 0.01, ****P ≤ 0.0001.

To study cross-reactive humoral immunity between coronaviruses pathogenic to humans, we examined IgG responses to spike of the eCoVs (HCoV-OC43, HCoV-HKU1, HCoV-229E and HCoV-NL63), SARS-CoV-1 and MERS-CoV in convalescent sera. Interestingly, although seronegative and seropositive individuals with prior mild/asymptomatic disease had comparable antibody titers against the eCoVs, convalesced patients with severe disease showed higher reactivity to spike trimer, but not or less to the S1 subunit, of HCoV-229E and HCoV-HKU1 (**Figures 2A, C**). Similar results were found for SARS-CoV-1 and MERS-CoV (**Figures 2E, F**). In the case of HCoV-OC43, antibody titers in convalesced patients with severe disease were only increased compared to mild/asymptomatic individuals, but not seronegative controls (**Figure 2D**). No difference was observed against HCoV-NL63 (**Figure 2B**). Predominance of elicited anti-spike trimer in comparison to S1-specific responses is likely the results of cross-reactivity induced by a structurally conserved S2 domain that shares substantial sequence homology between coronaviruses, in particular the *Betacoronaviruses (* 21). Since there has not been community transmission of SARS-CoV-1 and MERS-CoV in the Dutch population, these raised titers are probably due to cross-reactive SARS-CoV-2 antibodies. The higher titers to HCoV-OC43, HCoV-HKU1 and HCoV-229E on the other hand could reflect a history of exposure to these eCoVs, leading to a back boosting effect of B memory recall responses (16, 22, 23). A recent history to eCoV exposure could not be proven, as this requires sampling of these patients prior to development of COVID-19. When ranking sera from hospitalized patients during the acute phase of disease on their reported day of symptom onset, using the information from self-reported questionnaires, IgG titers against eCoVs were nearly a 10-fold higher compared to SARS-CoV-2 (**Supplementary Figure 2**). Although titers against eCoVs of the genus *Alphacoronavirus* (HCoV-229E and HCoV-NL63) were stable over the course of a month after symptom onset, eCoVs of the genus *Betacoronavirus* (HCoV-HKU1 and HCoV-OC43) were slowly rising, which can be explained by presence of a back boosting effect.

### Anti-Spike IgG Titers Against eCoVs of the Genus Betacoronavirus Correlate in Patients With Fatal COVID-19

We subsequently investigated potential immune correlates in patients with acute severe COVID-19 at the moment of hospital admission, based on clinical outcome. IgG titers between patients admitted to the ICU (severe disease) and patients admitted to the pulmonary ward (moderate disease) did not significantly differ (**Supplementary Figure 3**). We then analyzed SARS-CoV-2 and eCoV-specific IgG in subgroups stratified by disease outcome, comparing survivors (non-fatal disease) and non-survivors (fatal disease). Median responses to spike trimer were lower in patients with fatal disease, although this was not significantly different, although this was not significant. Of note, 53% and 42% of patients with fatal disease had undetectable titers against nucleocapsid and spike trimer protein respectively, below the detection thresholds of 10 AU/ml, compared to 25% and 15% of patients with non-fatal disease (**Supplementary Figure 4**). This may be due to delayed kinetics of serum antibody responses that are known to correlated with impaired viral control in deceased COVID-19 patients (24).

**Figure 3:**
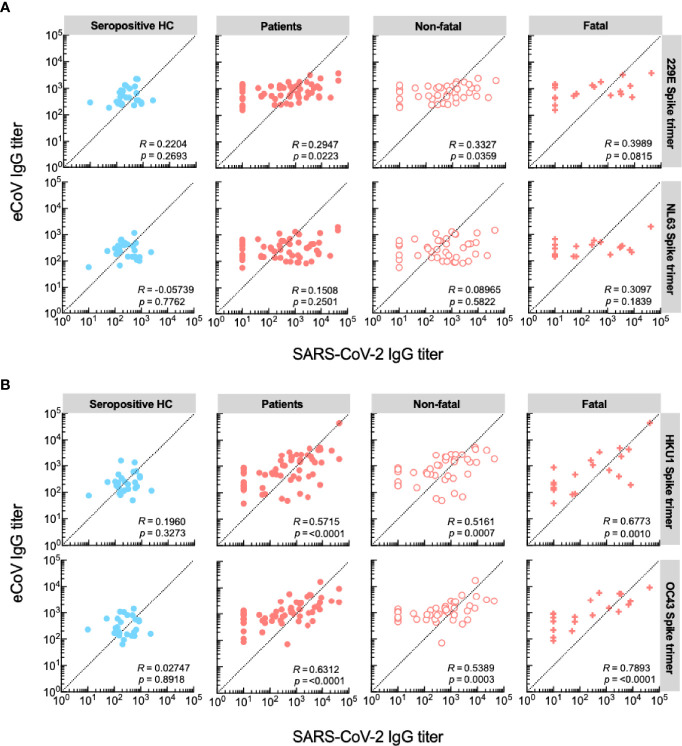
IgG titers against eCoVs of the genus Betacoronavirus correlate in patients with a fatal disease outcome. **(A)** Correlations between serum IgG responses against SARS-CoV-2 and eCoVs of the genus Alphaviruses. **(B)** Correlations between serum IgG responses against SARS-CoV-2 and eCoVs of the genus Betaviruses. Spearman’s rank correlations are shown for each pair of spike trimer antigens, presented as R value and significance.

Recently a pre-print has been published in which the authors describe that IgG responses to the spike protein of SARS-CoV-2 and eCoVs of the genus *Betacoronavirus* correlated in COVID-19 patients with a fatal outcome, which was mapped to a shared epitope on the S2 region (25). To test this hypothesis in our cohort, we performed a similar analysis and calculated Spearman’s rank correlations of anti-spike trimer antibody titer pairs in the cohorts based on outcome (non-fatal VS fatal). We also included a group of seropositive healthy controls. There was a correlation of moderate strength between anti-SARS-CoV-2 titers and titers to eCoVs of the genus *Betacoronavirus*, but not *Alphacoronavirus*, in hospitalized patients, whereas no such correlation was seen amongst seropositive healthy controls (**Figures 3A, B**). Interestingly, patients with fatal disease primarily contributed to this correlation (R values: 0.68 and 0.79 for HCoV-HKU1 and HCoV-OC43, respectively).

Taken together, these results indicate that patients with severe disease, who mount high IgG anti-SARS-CoV-2 responses also mount a response to eCoVs of the genus *Betacoronavirus*, which is likely not aimed at the S1 subunit. Correlation of anti-spike trimer titers, which were predominantly observed during fatal COVID-19, support presence of a back boosting effect that does not protect from a fatal disease course.

### Secretory IgA to eCoVs Is Detected Mainly in Patients With Severe COVID-19

Mucosal IgA provides a typical correlate of protection to various respiratory viruses and has been shown to play a role in our immune defenses during early SARS-CoV-2 infections (26). High salivary IgA specific to SARS-CoV-2 is present weeks after the onset of symptoms, although the durability is still unclear (27). We assessed IgA reactivity to SARS-CoV-2 and eCoVs in saliva of a set of patients of whom we were able to collect sufficient saliva for subsequent analysis of IgA, at the time of hospital admission to the pulmonary ward or ICU. We also included a subset of healthy SARS-CoV-2 seropositive controls. Since secretory IgA levels vary extensively across the day, we did not quantify IgA, but instead reported whether reactivity was present (28).

**Figure 4:**
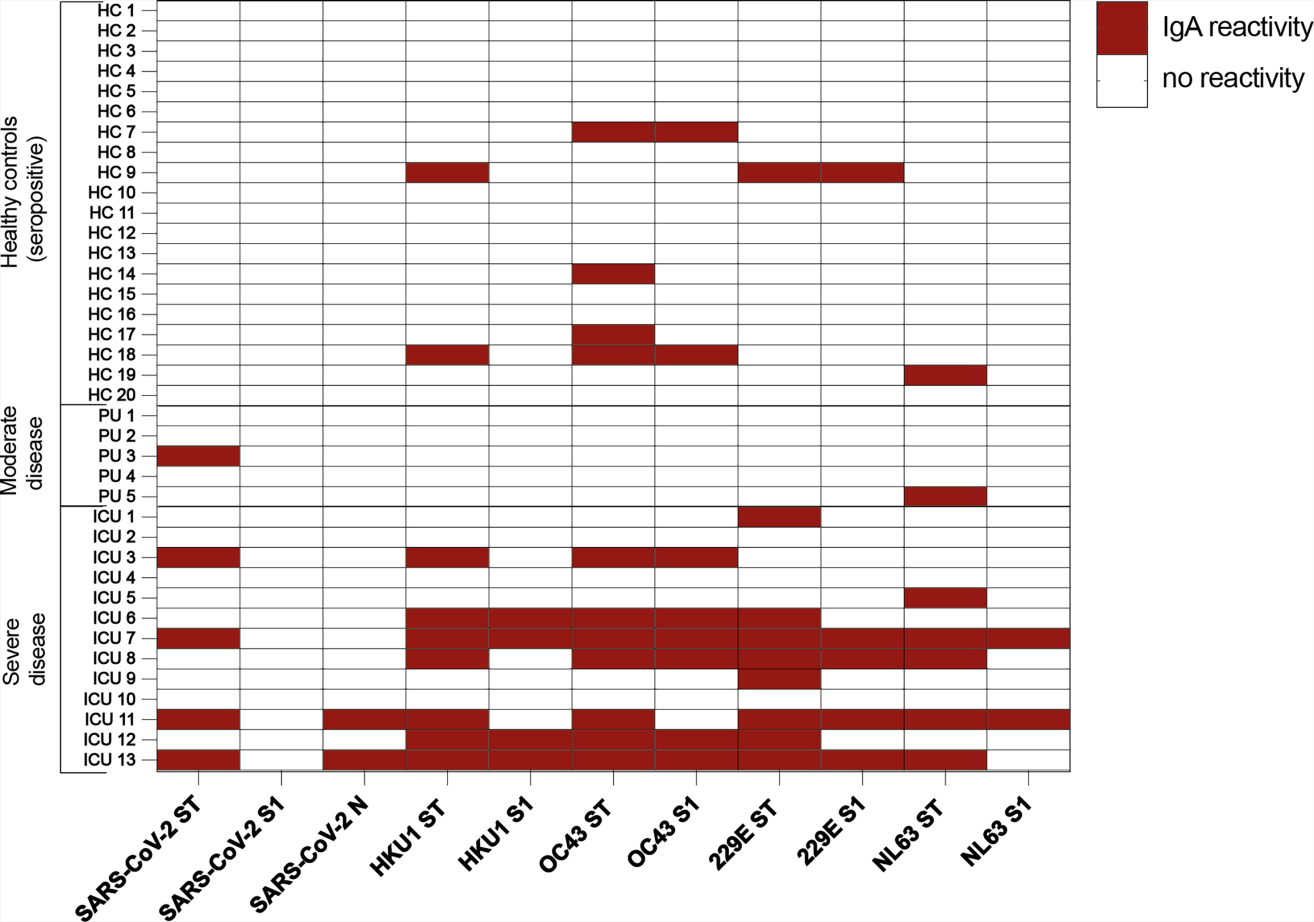
Positive IgA responses detected in saliva of patients with severe COVID-19. Heatmap visualization of IgA antibody titers against recombinant trimeric spike proteins and monomeric S1 subunits of SARS-CoV-2 and eCoVs measured by protein microarray in serially diluted saliva samples of COVID-19 patients with moderate and severe disease compared to seropositive healthy controls. Positive responses are indicated by red squares. ICU, intensive care unit; PU, pulmonary unit.

There was no correlation between time from symptom onset to sampling. Anti-spike IgA specific for SARS-CoV-2 was only detected against the S2-containing trimer in 31% (4/13) of the ICU subset, comprising of patients in need of invasive^nbsp;mechanical ventilation or critical care due to disease complications (**Figure 4**). Interestingly, IgA reactivity against one or multiple eCoVs was observed in 10/13 (77%) of patients with severe disease, which was a significantly higher proportion than the 1/5 (20%) seen in moderate disease controls (*p* = 0.05), as well as compared to 5/20 (25%) seropositive healthy controls (*p* = 0.005). These IgA responses targeted both the full spike trimer and the S1 subunit of each of the eCoVs. Thus, based on this proxy measurement in saliva of a small cohort of patients, presence of mucosal secretory IgA against spike protein of eCoVs may be an immune correlate of severe disease, and further supports that heterologous responses in patients with severe COVID-19 are not cross-protective.

## Discussion

Here, we studied heterologous immune responses using a protein microarray to measure IgG and secretory IgA directed against the spike protein of SARS-CoV-2 and eCoVs in COVID-19 patients during the acute and convalescent phase of disease. We demonstrated significantly higher IgG levels against the spike protein of eCoVs during convalescence upon severe COVID-19 compared to mild/asymptomatic infections. Reactivity was mainly observed against the S2-containing spike trimer of *Betacoronaviruses* (HCoV-OC43, HCoV-HKU1, and SARS-CoV-1 OC43, HKU1, and SARS-CoV-1), although anti-spike S1 titers were also slightly higher, which may be due some degree of homologous epitopes shared between these related viruses (29, 30). Altogether, our findings supports the presence of non-protective cross-reactivity, potentially due to epitope similarity in evolutionarily conserved S2 regions of spike, in line with results from multiple recent publications (13–15, 31). Although we did not perform neutralization assays, a recent report addressed this issue in a similar disease cohort, and concluded that back boosted antibodies elicited by B cell memory recall against eCoVs were not effective in neutralization of the SARS-CoV-2 virus (23). Thus, we hypothesize that back boosted heterologous antibodies are non-protective, and potentially contribute to, an unfavorable disease course.

Certain limitations must be mentioned in relation to our findings. First of all, our study design did not enable us to assess titers of patients before they contracted a SARS-CoV-2 infection, limiting the possibility to infer causality. Interestingly, a recent prospective analysis demonstrated that individuals with higher anti-eCoV IgG and IgA baseline levels were associated with increased SARS-CoV-2 antibody levels which correlated with greater disease severity (32). Secondly, patients with fatal COVID-19 were significantly older in our study and could therefore have been exposed more frequently to infections with eCoVs (33). However, protective antibodies are known to be short-lasting and wane rapidly over the course of a year (34). It thus remains unclear if these heterologous responses reflect recent re-exposure to a specific eCoV or back booster clones of a robust long-term B cell memory compartment shaped by life-long exposure to these viruses (23). Furthermore, there could be an underlying hyperinflammatory immune-system condition which induces exuberant polyreactive responses in patients with a fatal outcome (35). However, based on patient histories we had no indications that such conditions were present. Finally, it should be mentioned that classification based on hospital department admission does not necessarily reflect disease severity directly linked to COVID-19, since pre-existing comorbidities may also dictate the disease course. Most patients however, were initially admitted to the ICU due to respiratory distress from COVID-19 pneumonia.

A novel finding of this study was the presence of secretory mucosal IgA reactive to spike protein of eCoVs, in particular the eCoVs belonging to the same genus as SARS-CoV-2, in patients with severe COVID-19. Whilst the number of samples available was limited, we did not observe such degree of eCoV-specific IgA reactivity in patients with moderate disease. Whether this is a result of recent eCoV re-exposure, or sign of a polyreactive IgA response due to excessive immune activation, is an interesting question that remains to be addressed. Of note, IgA responses to the SARS-CoV-2-specific spike proteins were largely undetectable.

Based on our results and data from recent studies (16, 22, 23, 32), we speculate that such heterologous responses to eCoVs might be a sign of immune imprinting. This phenomenon has been demonstrated in relation to influenza virus infections and some flavivirus infections, in which exposure to antigen of a novel but similar naturally drifted, or vaccine-derived, strain elicits an ineffective memory recall response to the previous strain, delaying or attenuating efficient neutralization (36). To address involvement of immune imprinting as a deleterious pathogen-host interaction, further experiments should be conducted to assess if back boosted antibodies and memory B cells against seasonal coronaviruses may hinder the affinity of neutralizing antibodies against SARS-CoV-2, and how this affects breadth, depth, and functionality of the immune repertoire during an acute infection. The role of mucosal IgA herein remains speculative, and a mechanism by which SARS-CoV-2 viral entry into mucosal epithelium may be facilitated by plasmablasts-derived antibody-mediated cross-recognition of heterotypic secretory IgA, may be considered.

## Methods

### Ethics Committee Approval

The regional Medical Research Ethics Committees United approved the study (Nieuwegein, the Netherlands; MEC-U: NL73618.100.20).

### Study Cohorts

Patients who were admitted to the Diakonessenhuis hospital from May 2020 suspected with COVID-19 disease were enrolled in the SIR study once SARS-CoV-2 infection was confirmed with PCR on nasal mucosa or bronchial excrete. Patients were either included from the pulmonary ward or directly from the ICU, and no exclusion criteria were used. Medical history was retrieved from the medical records that was accessible. Healthy controls consisted of hospital personnel, without a medical history of a condition that could be expected to influence their immune status or the natural history of COVID-19 disease, who were asked to voluntarily participate in the study. For validation of the microarray platform, an extra set of healthy seronegative individuals (hospital personnel) was added who were vaccinated with the first shot (n=8) or the booster (n=4) of the BioNTech/Pfizer mRNA vaccin (BNT162b2).

### Protein Expression and Microarray Analyses

Secreted recombinant protein were produced in-house by transfection of plasmid DNA in mammalian HEK293F suspension cells as previously described (37). The transfected pPPI4 plasmids contained a sequence encoding for the corresponding antigen, followed by a HIS-tag. The following antigens were used: spike protein trimer and S1 subunit of SARS-CoV-2 (GenBank QHD43416.1), eCoV HCoV-229E S1 (GenBank JX503061.1), eCoV HCoV-HKU1 S1 (GenBank ADN03339.1), eCoV HCoV-OC43 S1 (GenBank AIX10763), eCoV HCoV-NL63 S1 (GenBank ABE97130.1) and MERS-CoV S1 (GenBank KJ650297.1). In case of spike trimer proteins, the HIS-tag was preceded by a trimerization motif. Here, a prefusion-stabilized S protein ectodomain of SARS-CoV-2 and SARS-CoV with a T4 trimerization domain and hexahistidine (His) tag was designed as previously described (31). The same procedure was used to generate prefusion-stabilized S proteins of MERS-CoV, hCoV-229E, hCoV-NL63, and hCoV-OC43. More detailed information on the peptide sequences can be found in a previous report (31).

Secreted recombinant protein was purified from the cell suspension by gravity flow chromatography using Ni-Nta beads. For spike trimer proteins, monomers and dimers were excluded from the mixture using size exclusion chromatography. For spike S1, dimerization and complex formation was excluded. The nucleocapsid protein of SARS-CoV-2 was obtained from a commercial source (Sino Biological, Eschborn, Germany; Cat: 40588-V08B). Preparation of human coronavirus protein micro-array was based on previously described methodology (19).

### IgG Measurement in Serum

Sera were tested in 8 3-fold dilutions starting at 1:10, diluted in Blotto buffer containing 0.1% Surfact-Amps20 (ThermoFisher). Goat anti-human IgG, F(ab’)2 fragment specific, Alexa Fluor 647-conjugated (Jackson Immuno Research, West Grove, USA) was used in a 1:1000 dilution in Blotto buffer containing 0.1% Surfact-Amps20. IgG experiments on sera were performed once, divided in five batches. A pool of SARAS-CoV-2 positive control samples with known titers to eCoVs and SARS-CoV-2, were included in every run to insure low inter-assay variation.

### IgA Measurement in Saliva

If possible, patient saliva was collected and immediate stored in -80°C awaiting further use, unless lack of production made it impossible or too low yields were obtained. As many samples as possible were collected, without specifically defined selection criteria. Saliva samples were tested in 4 3-fold dilutions starting 1:2, diluted in Blotto buffer containing 0.1% Surfact-Amps20 (ThermoFisher). Goat anti-human IgA, α chain, F(ab’)2 fragment specific, Alexa Fluor 647-conjugated (Jackson Immuno Research, West Grove, USA) was used in a 1:500 dilution in Blotto buffer containing 0.1% Surfact-Amps20. IgA experiments on saliva were performed once, divided in two batches (with inclusion of control samples with known reactivity).

### Wantai Ab ELISA

The presence of anti-SARS-CoV-2 antibodies in serum was determined by using the Wantai SARS-CoV-2 ELISA (Wantai Biological Pharmacy, Beijing, China). The assay was performed according to the manufacturer’s protocol, using a DS2-automated ELISA instrument (Dynex Technologies, Chantilly, VA, USA) and analyzed with the DS-Matrix™ software (Dynex Technologies). The assay is based on a double-antigen sandwich principle that detects total antibodies binding SARS-CoV-2 spike protein receptor binding domain (RBD) in human serum or plasma. The results are expressed as ratios of the cut-off. The cut-off value was calculated according to the manufacturer’s instruction by adding the mean of three negative controls (minimum 0.03) to 0.16. Results with a ratio greater than 1 are considered positive.

### Statistical Analysis

Statistical values (ROC curve, Spearman’s rank correlations, and *p* values) were calculated with GraphPad Prism version 9.1.0. Calculation details on the ROC and threshold can be found on: https://www.graphpad.com/guides/prism/latest/statistics/stat_calcualtion_details_for_roc_cu.htm. Groups were compared with unpaired one-way ANOVA or Mann–Whitney U test for comparing two or more groups respectively.

## Data Availability Statement

The original contributions presented in the study are included in the article/**Supplementary Material**. Further inquiries can be directed to the corresponding author.

## Ethics Statement

The studies involving human participants were reviewed and approved by The regional Medical Research Ethics Committees United (Nieuwegein, the Netherlands; MEC-U: NL73618.100.20). The patients/participants provided their written informed consent to participate in this study.

## Author Contributions

SvT performed the protein microarray experiments, and G-JG and SS were involved in production of recombinant proteins. FV, SG, and AB assisted with sample collection. WS and SW were involved with data analysis and presentation. WS wrote the manuscript, with support from DE, MH, and ST. All authors contributed to the article and approved the submitted version.

## Conflict of Interest

The authors declare that the research was conducted in the absence of any commercial or financial relationships that could be construed as a potential conflict of interest.

## Publisher’s Note

All claims expressed in this article are solely those of the authors and do not necessarily represent those of their affiliated organizations, or those of the publisher, the editors and the reviewers. Any product that may be evaluated in this article, or claim that may be made by its manufacturer, is not guaranteed or endorsed by the publisher.

## Supplementary Material

Supplementary Figure 1 organization chart of disease and control groups. Organization chart showing the different groups that were defined to assess the acute and convalescent phase of immune responses to SARS-CoV-2, endemic, and emerging coronaviruses.

Supplementary Figure 2 IgG antibody titers in COVID-19 patients ranked based on days after symptom onset. **(A)** IgG antibody titers against recombinant trimeric spike proteins human coronaviruses measured by protein microarray in sera presented as mean value with SEM. **(B)** Data from each protein antigen plotted separately with individual data points per time point (one-way ANOVA, Šidák multiple comparison test).

Supplementary Figure 3 IgG antibody responses in patients with moderate and severe disease. IgG antibody titers against recombinant trimeric spike proteins and monomeric S1 subunits of human coronaviruses measured by protein microarray in sera from pulmonary ward with moderate disease (yellow box) and ICU with severe disease (red box) COVID-19 patients. Median titers are presented (min and max value).

Supplementary Figure 4 IgG antibody responses in patients with non-fatal and fatal disease. IgG titers were measured against recombinant trimeric spike proteins and monomeric S1 subunits of human coronaviruses by protein microarray in sera from acutely ill patients of the ICU with non-fatal and fatal disease. Data are represented as the median value (min and max value), significance (Mann-Whitney U test) *P < 0.05.
