# Supplementary figures and images for: Heterologous Immune Responses of Serum IgG and Secretory IgA Against the Spike Protein of Endemic Coronaviruses During Severe COVID-19
Source: Front Immunol. 2022 Mar 9;13:839367. doi: 10.3389/fimmu.2022.839367 (PMC8959642; doi:10.3389/fimmu.2022.839367)

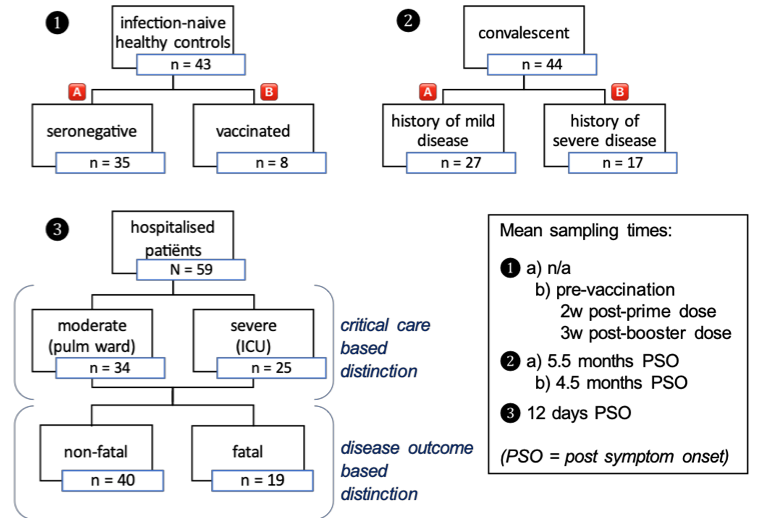

Supplement: Supplementary Figure 1 — organization chart of disease and control groups. Organization chart showing the different groups that were defined to assess the acute and convalescent phase of immune responses to SARS-CoV-2, endemic, and emerging coronaviruses. [file Image_1.tiff]

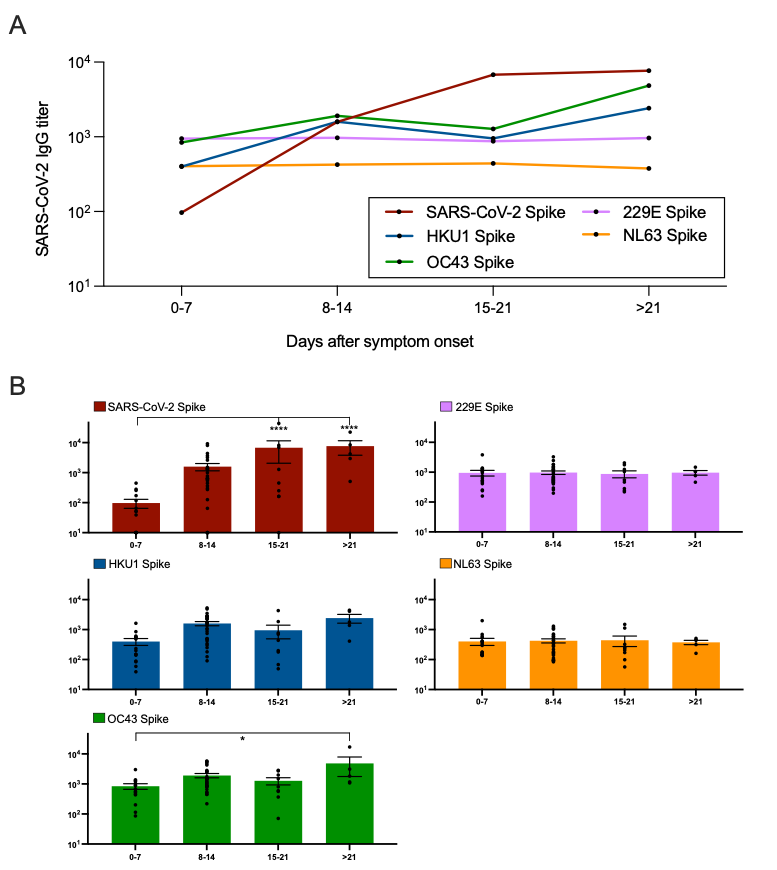

Supplement: Supplementary Figure 2 — IgG antibody titers in COVID-19 patients ranked based on days after symptom onset. (A) IgG antibody titers against recombinant trimeric spike proteins human coronaviruses measured by protein microarray in sera presented as mean value with SEM. (B) Data from each protein antigen plotted separately with individual data points per time point (one-way ANOVA, Šidák multiple comparison test). [file Image_2.tiff]

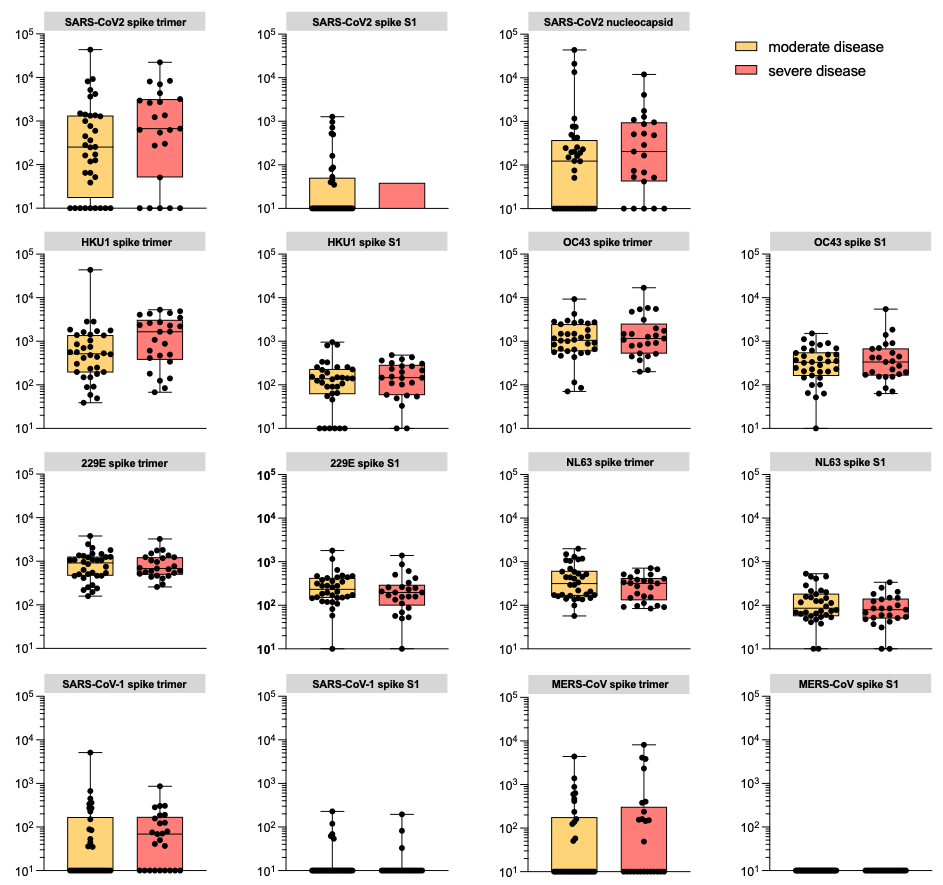

Supplement: Supplementary Figure 3 — IgG antibody responses in patients with moderate and severe disease. IgG antibody titers against recombinant trimeric spike proteins and monomeric S1 subunits of human coronaviruses measured by protein microarray in sera from pulmonary ward with moderate disease (yellow box) and ICU with severe disease (red box) COVID-19 patients. Median titers are presented (min and max value). [file Image_3.tiff]

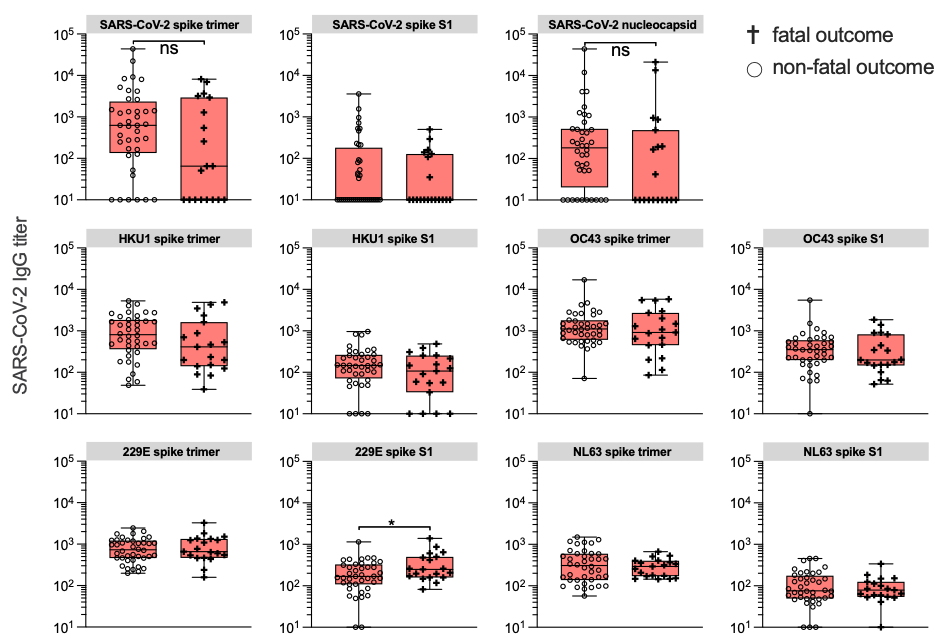

Supplement: Supplementary Figure 4 — IgG antibody responses in patients with non-fatal and fatal disease. IgG titers were measured against recombinant trimeric spike proteins and monomeric S1 subunits of human coronaviruses by protein microarray in sera from acutely ill patients of the ICU with non-fatal and fatal disease. Data are represented as the median value (min and max value), significance (Mann-Whitney U test) *P < 0.05. [file Image_4.tiff]
